# Febrile ulceronecrotic Mucha-Habermann disease: a case report and literature review in preschool-aged children

**DOI:** 10.3389/fped.2026.1762372

**Published:** 2026-04-23

**Authors:** Jun Luo, Xueshuang Liu, Xuan Liu, Kai Wang, Wenshuang Wei

**Affiliations:** 1 Ministry of Basic Medical Education, Dazhou Vocational College of Chinese Medicine, Dazhou, China; 2 Department of Dermatology and Venereology, Maternal and Child Health Hospital of Hubei Province, Wuhan, China

**Keywords:** febrile ulceronecrotic Mucha-Habermann disease, intravenous immunoglobulin, lymphoproliferative disorder, methotrexate, pityriasis lichenoides et varioliformis acuta, prednisolone, preschool children

## Abstract

Febrile Ulceronecrotic Mucha- Habermann Disease (FUMHD) is a rare and severe specialized type of Pityriasis Lichenoides Et Varioliformis Acuta (PLEVA). It is characterized by rapid onset of painful necrotic skin lesions and systemic symptoms. The diagnosis of FUMHD is complex and is easily misdiagnosed in the early stages. Despite the increasing number of reports of FUMHD in recent years, it is still uncommon in preschool (3–6 years) children. We here report a 3-year-9-month-old girl of FUMHD from China, cured through combined antibiotics, intravenous immunoglobulin, methotrexate, and prednisone. Besides, a literature review was conducted to synthesize the key findings of preschool FUMHD cases.

## 1 Introduction

Febrile ulceronecrotic Mucha-Habermann disease (FUMHD) is recognized as a distinct variant of pityriasis lichenoides et varioliformis acuta (PLEVA). Common clinical manifestations of FUMHD encompass erythematous patches, scaly papules, and ulceronecrotic lesions, typically accompanied by persistent fever, hemorrhagic features, mucosal involvement, and secondary infections (1, 2). Although FUMHD is a rare disorder, it carries the potential for progression from a mild initial presentation to severe complications, including life-threatening systemic involvement or even fatal outcomes (1). The diagnosis of FUMHD is intricate, hinging on characteristic clinical manifestations, distinctive histopathological findings, and rigorous exclusion of other disorders such as PLEVA, chickenpox, or Steven-Johnson Syndrome (3–7).

This article reports a case of a 3-year-9-month-old girl who achieved complete remission through a combination regimen of intravenous antibiotics, intravenous immunoglobulin (IVIG) pulse therapy, methotrexate, and prednisone. Since first described in 1966, over 100 FUMHD cases have been globally reported, with the ages ranging from 8 months to 82 years (8–10). While previous literature has conducted comprehensive evaluations of FUMHD, existing systematic reviews predominantly focus on disease presentation and therapeutic outcomes in children over six years old. The clinical characterization and management of younger populations, particularly preschool-aged children, remain insufficiently addressed, highlighting a critical gap in age-specific evidence (2). To better characterize the disease profile in this population, a systematic literature review was conducted on previously reported cases of FUMHD in preschool-aged children (3–6 years).

## 2 Case report

### 2.1 Clinical presentation and differential diagnosis

The patient, a 3-year-9-month-old girl, developed red plaques on her trunk and limbs without obvious cause 5 months ago, which later formed scabs. Before presentation to us, she was empirically diagnosed with “psoriasis” or “parapsoriasis” and had received topical medication. One week later, her condition significantly worsened with increased erythema, erosions, and high fever, and failed to respond to the medication, which prompted her transfer to our hospital for treatment.

**Figure 1:**
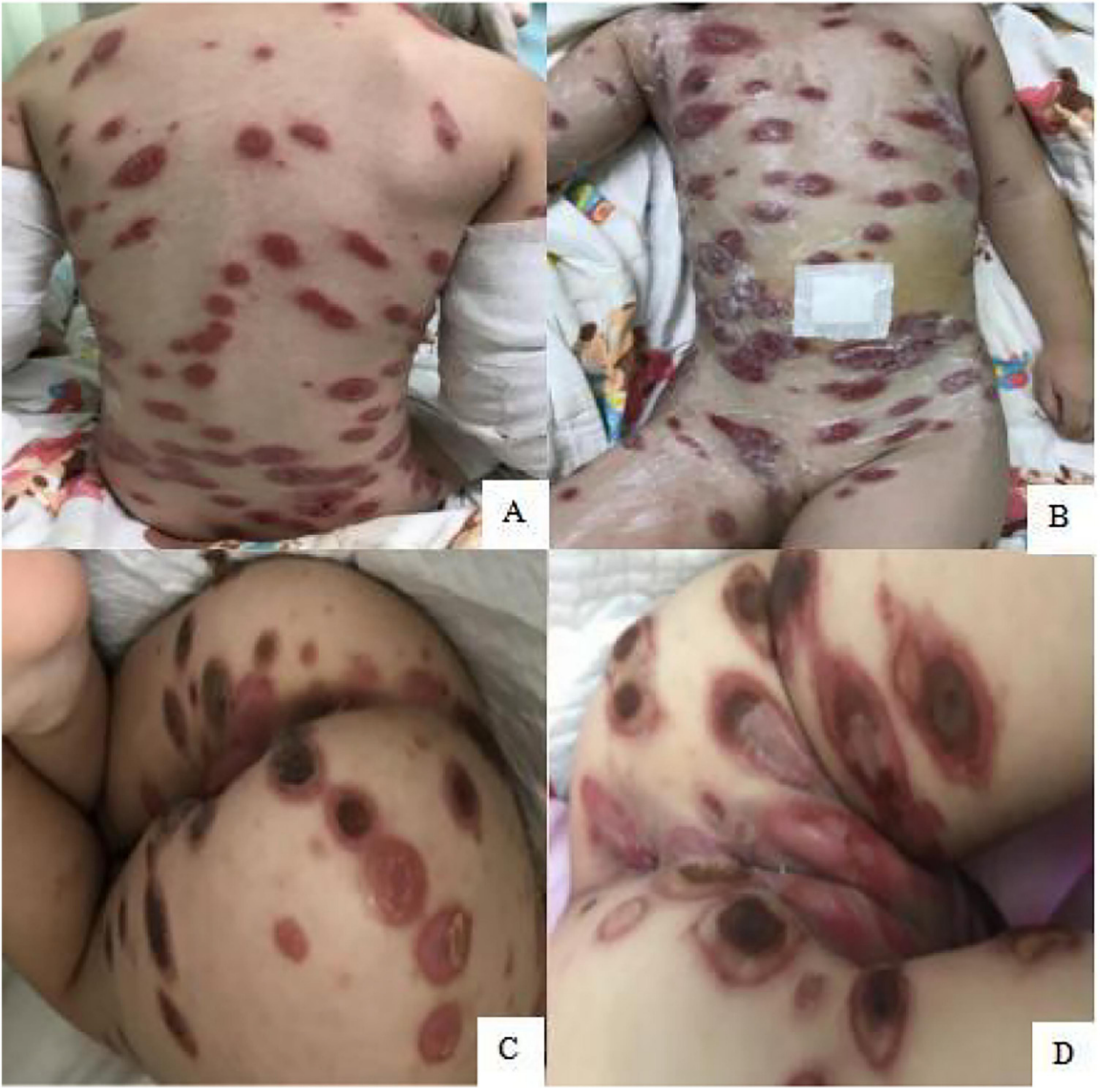
**(A–D)** Scattered large dark red patches on the trunk and extremities on hospital day 1.

Cutaneous examination revealed widespread oval, dark red, proliferative plaques measuring 1–6 cm on the child's limbs and trunk, surrounded by bright red borders with well-defined margins and no coalescence. Some plaques showed central ulceration with surface exudation, and scattered papules were observed on the limbs (Figure 1). Oral mucosal examination demonstrated erosions on the hard palate, bilateral buccal mucosa, tongue surface, and base of the tongue. The genitalia and buttocks exhibited dark red plaques of varying sizes with clear boundaries, showing central necrosis and crusting, with some ulcerated areas displaying exudate (Figure 1). Upon admission, the child had a body temperature of 38 °C and a heart rate of 95 bpm. Cardiopulmonary examination was unremarkable, and no enlargement of superficial lymph nodes was detected throughout the body.

Based on the patient's symptoms and physical examination findings, the following differential diagnoses were considered at admission: (1) severe drug hypersensitivity reactions, including Stevens-Johnson syndrome (SJS)/toxic epidermal necrolysis (TEN) and severe erythema multiforme (EM major); (2) infection; (3) atypical Kawasaki disease; (4) autoimmune bullous disease; (5) lymphoma; (6) pyoderma gangrenosum.

#### 2.1.1 Exclusion of SJS/TEN and EM major

Despite morphological similarities (target-shaped lesions measuring 1–6 cm, with central ulceration and peripheral vesicles), the following clinical and pathological features support the exclusion of these diagnoses:

Course of disease: The course of this disease lasted 5 months and followed a relapse-remission pattern, in sharp contrast to the acute onset of SJS/TEN (typically within 4 weeks of drug exposure) (11) and the self-limiting nature of EM major (2–4 weeks, mean 15.9 days) (12).

Histopathological differences: Biopsy revealed focal epidermal necrosis with interface dermatitis and leukocytoclastic vasculitis (destruction of blood vessel walls), rather than the full-thickness epidermal necrosis and widespread keratinocyte apoptosis typical of SJS/TEN (11), or the vacuolar interface dermatitis with target-shaped lesions characteristic of EM major (12).

Exclusion of triggers: A detailed medication history revealed no exposure to high-risk drugs associated with SJS/TEN. Routine infection screening (including viral PCR and EBV-DNA) was negative, effectively ruling out common infectious triggers of EM major (herpes simplex virus and Mycoplasma infections) (12). It is worth noting that the original medical records did not document specific PCR test results for herpes simplex virus (HSV), varicella-zoster virus (VZV), or Mycoplasma pneumoniae. Such tests are targeted screening tools for infectious causes of EM major, and serological testing for Mycoplasma pneumoniae IgM antibodies and HSV/VZV antibodies was also not performed in this case.

Laboratory investigations revealed elevated lymphocytes, monocytes, C-reactive protein (CRP), and serum amyloid A (SAA), while white blood cell count and neutrophils remained within normal limits. Peripheral blood smears were unremarkable, and blood cultures were negative; however, skin swab cultures grew Staphylococcus aureus subsp. aureus. Liver/kidney function tests, electrolytes, endotoxin, procalcitonin, and humoral immunity panels were all normal. Infectious disease screening (tuberculosis antibodies, syphilis, HIV, fungal/viral PCRs, and EBV-DNA) returned negative results. Imaging studies (chest CT, cardiac ultrasound) and bone marrow aspiration showed no abnormalities. Direct immunofluorescence (IgG, C3, IgA, IgM) was negative, as were immunohistochemical stains (TIA1, CD30, CD20, CD79a, CD1a). However, CD3, CD4, CD8, and CD56 were positive, with focal S-100 and CD68 positivity. TCR gene rearrangement analysis detected polyclonal bands. Detailed laboratory results were shown in Table 1.

**Table 1:** Clinical laboratory report.

Test name	Result	Flags
Leukocytes	9.50 × 10⁹/L	Normal
Neutrophils	4.27 × 10⁹/L	Normal
Lymphocytes	3.82 × 10⁹/L	High
Lymphomonocytes	1.05 × 10⁹/L	High
C-reactive protein	62.00 mg/L	High
Serum amyloid protein	79.90 mg/L	High
Peripheral blood smear	Normal	Normal
Blood culture	Negative	Normal
Skin secretion culture	Staphylococcus aureus subsp. aureus	
Tuberculosis antibody	Negative	Normal
Syphilis	Negative	Normal
HIV	Negative	Normal
Fungi	Negative	Normal
Virus	Negative	Normal
EB-DNA	Negative	Normal
Chest CT	Negative	Normal
Cardiac color doppler ultrasound	Negative	Normal
Bone marrow puncture	Negative	Normal
Liver and kidney function	Normal	Normal
Electrolytes	Normal	Normal
Endotoxin	Normal	Normal
Procalcitonin	Normal	Normal
Humoral immune function tests	Normal	Normal

### 2.2 Pathology results

**Figure 2:**
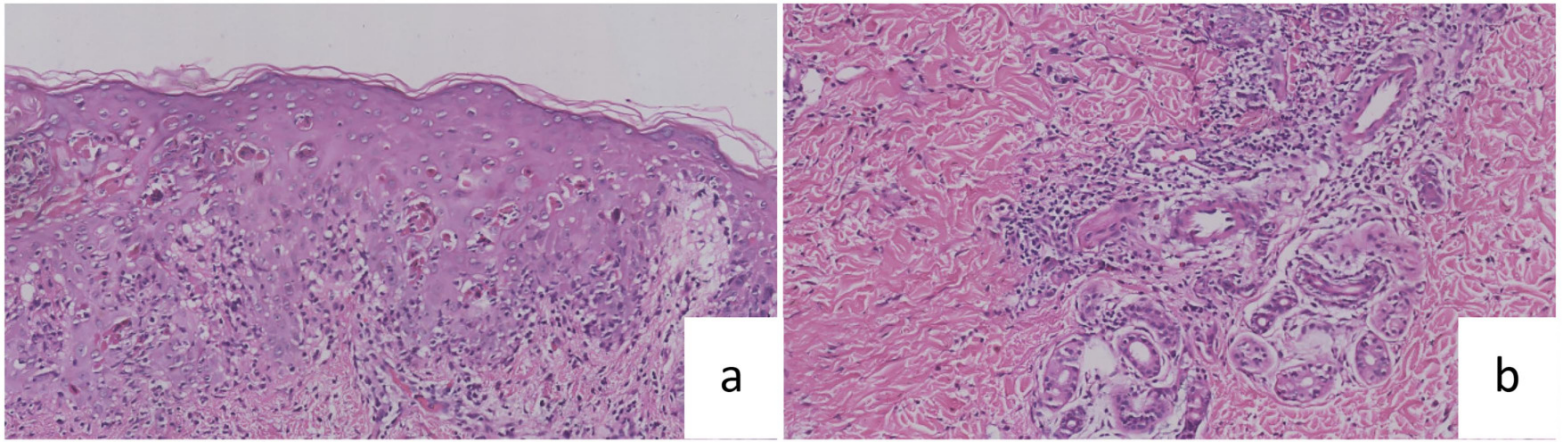
**(a, b)** Epidermal necrosis, superficial crusting, a large number of necrotic keratin-forming cells, banded infiltration of lymphoid histiocytes in the superficial dermis, infiltration of lymphoid and histiocytic eosinophils and mast cells in the superficial and deep layers of blood vessels, around the appendages, and among the collagen, and destruction of the vascular wall was seen.

The histopathological findings (Figure 2) revealed epidermal necrosis with superficial crust formation, featuring numerous necrotic keratinocytes. A band-like infiltrate of lymphocytes and histiocytes was observed in the superficial dermis, accompanied by perivascular, periadnexal, and interstitial infiltration of lymphocytes, histiocytes, eosinophils, and mast cells in both superficial and deep dermal layers. To rule out autoimmune bullous diseases and malignant lymphatic tumors, direct immunofluorescence and immunohistochemical examinations were subsequently performed. The direct immunofluorescence results (IgG, C3, IgA, IgM) were all negative. Immunohistochemical staining revealed positivity for CD3, CD4, CD8, and CD56, with focal positivity for S-100 and CD68, while TIA1, CD30, CD20, CD79a, and CD1a were negative. Molecular analysis was performed to assess clonality; T-cell receptor (TCR) gene rearrangement analysis detected polyclonal bands. Additionally, vascular wall destruction was evident.

Based on the patient's symptoms and physical examination findings, the following differential diagnoses were considered upon admission: infection, atypical kawasaki disease, autoimmune bullous disease, lymphoma, pyoderma gangrenosum, drug-induced skin reaction. The child presented with systemic symptoms, but leukocyte levels were within normal range, effectively ruling out bacterial infection. Based on pathological findings and ancillary tests, atypical Kawasaki disease, autoimmune bullous disorders, lymphoma, and pyoderma gangrenosum were excluded. The disease course had persisted for over five months with no history of relevant medication use, making drug-induced skin reactions highly unlikely. Combined with the patient's clinical manifestations, physical examination, and laboratory results with the diagnostic criteria outlined in the referenced literature (4), the final diagnosis was established as febrile ulceronecrotic Mucha-Habermann disease (FUMHD).

**Figure 3:**
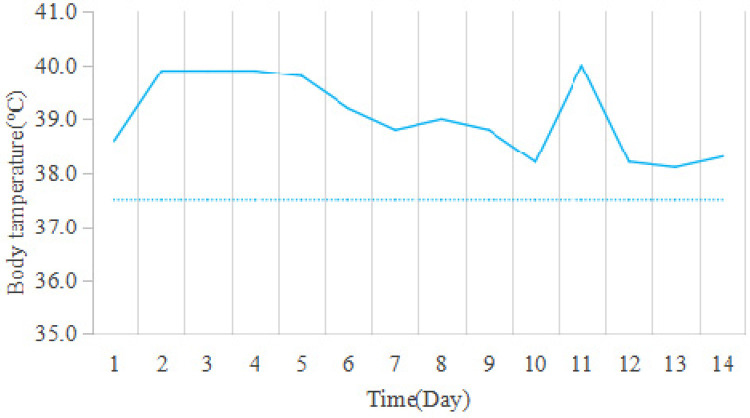
Body temperature.

**Figure 4:**
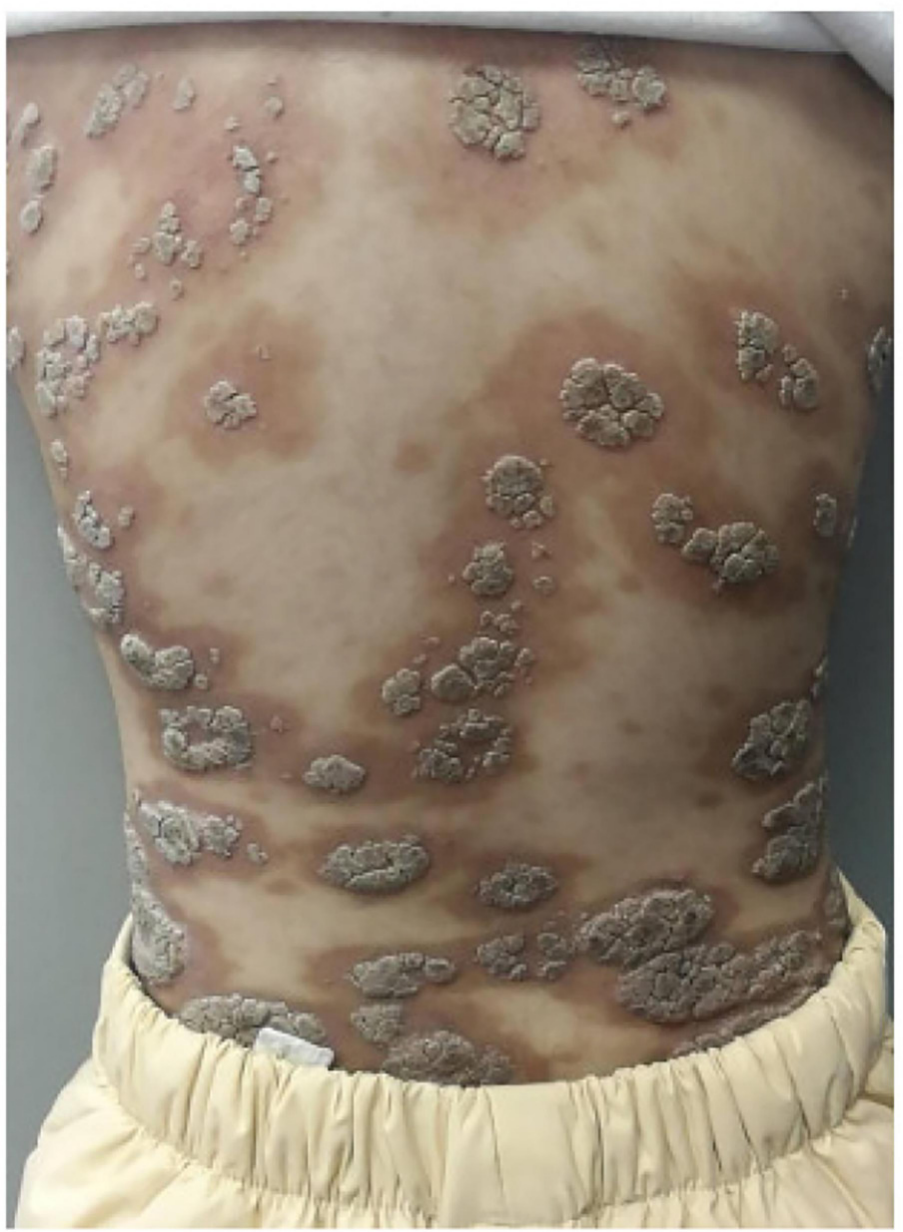
Darkening erythematous lesions, reduced exudation, formation of dry crusts after 2 weeks.

**Figure 5:**
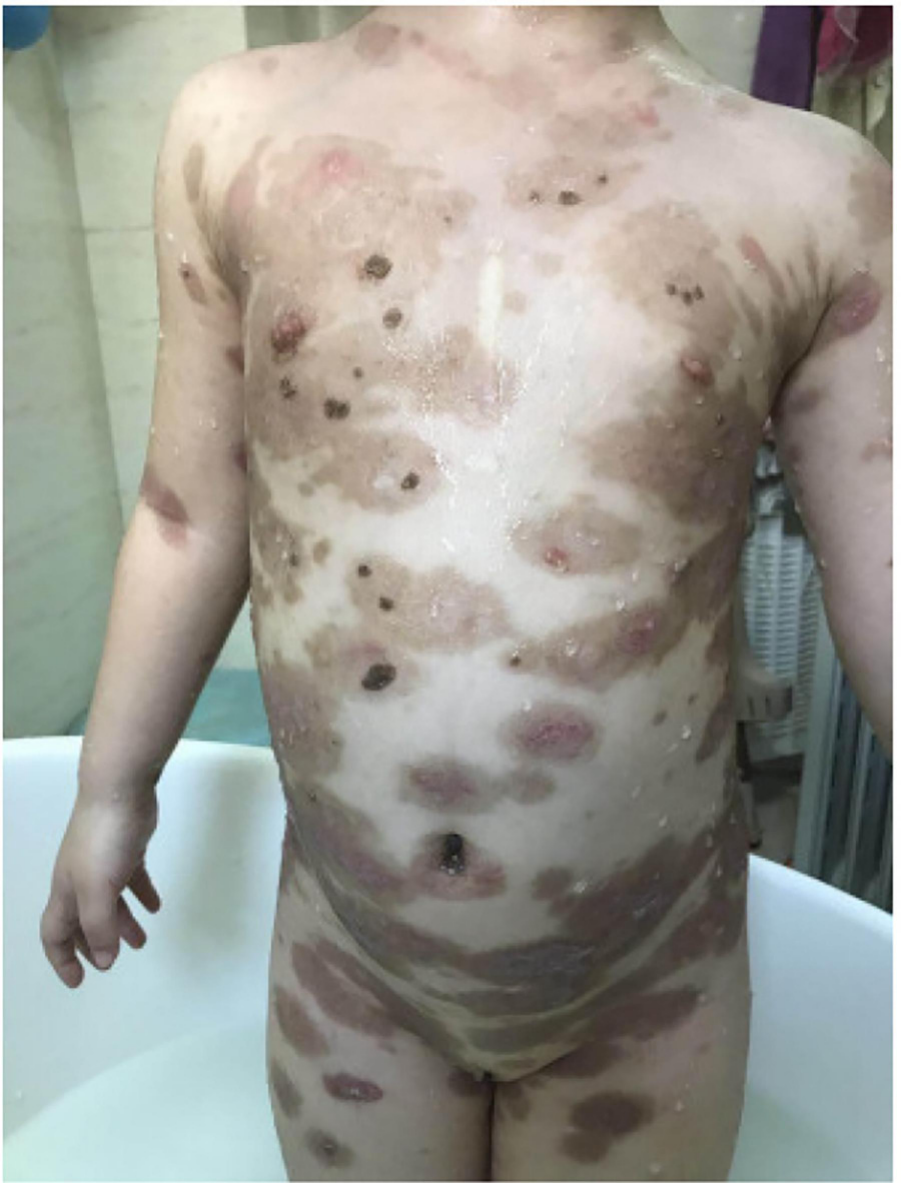
Nearly healed ulcerations, postinflammatory hyperpigmentation after 6 weeks.

The patient presented with persistent high fever, newly developed maculopapular rash, and hemorrhagic necrosis. Treatment was conducted in three distinct phases: Phase 1 (Days 1–3 of hospitalization): Following admission, the patient's temperature rose to 39.8 °C, and a skin swab culture tested positive for Staphylococcus aureus (secondary skin infection). Broad-spectrum intravenous antibiotics (cefmetazole, a second-generation cephalosporin, 5 mg/kg/ day, every 8 h) to control the bacterial infection and prevent progression to sepsis. Phase 2 (Days 3–7): Despite antibiotic therapy, the patient's fever persisted; therefore, intravenous immunoglobulin (IVIG, 1 g/kg/day for 2 consecutive days) was added as an immunomodulatory pulse therapy, and methylprednisolone (1 mg/kg/day) was administered to control acute inflammation. Phase 3 (from Day 7 onward): Once the condition stabilized, treatment was switched to oral prednisolone combined with methotrexate (MTX, initially 5 mg/day, later adjusted to 10 mg/m^2^ weekly) as maintenance therapy. Methotrexate is used as a first-line maintenance drug for long-term disease control and prevention of recurrence; this treatment strategy is consistent with previous literature reporting that methotrexate is the first-line drug for the long-term management of FUMHD in children (13). After initiation of combination therapy, the patient's temperature began to decline on Day 5, but remained initially above the normal range (Figure 3). However, the patient's condition gradually improved: the erythematous lesions darkened in color, exudation decreased, and dry crusts formed over the ulcerated areas (Figure 4). After 6 weeks of treatment, the ulcers had largely healed, leaving only post-inflammatory hyperpigmentation (Figure 5).

## 3 Discussion

### 3.1 Epidemiological and clinical characteristics of preschool FUMHD

Children aged 3–6 years represent a unique transitional period from early childhood to school age. Currently, reported cases of FUMHD in this age group remain limited. To our knowledge, only seven cases of FUMHD in preschool children have been documented, including the present case. Notably, three of these cases were initially misdiagnosed with other conditions such as psoriasis, viral infection, or impetigo prior to correct identification. This pattern highlights the diagnostic challenges and potential for misdiagnosis of FUMHD in this age group (Table 2). The current study comprehensively reviews all reported cases of FUMHD in 3–6 year-old, systematically analyzing epidemiological characteristics, clinical manifestations, diagnostic pitfalls, and therapeutic responses. Our findings aim to provide evidence to facilitate future research and improve clinical management in preschool children.

**Table 2:** Summary of clinical features and therapies in preschool-aged children of febrile ulceronecrotic Mucha-Habermann disease (FUMHD).

Reference	Age	Sex	Clinical symptoms	Immunological indicators	Therapy	Outcome
1 (14)	3 years	Male	Fever, maculopapular rash, lymphadenopathy, oral mucosal damage	Increase in CRP, erythrocyte sedimentation rate, leukocytes	ABS,	Cure
					IVIG,	
					ACV	
2 (15)	5 years	Female	Fever, papules, ulcerative plaques, swollen lymph nodes	Infiltration with CD3^+^/CD8^+^/TdT^−^, perivascular inflammation, elevated IL-2 receptor and elevated *α* (sIL-2Rα).	BAS,	Cure
					MTX,	
					SS,	
					VCR,	
					IVIG	
3 (16)	5 years	Male	Polymorphic rash (erythema/hemorrhagic macules/necrotizing crusted papules), high fever, seizures, mucosal damage	Pseudomonas aeruginosa infection	IVIG,	Cure
					SS,	
					MTX,	
					ABS,	
					ACV,	
					DDS	
4 (17)	5 years	Female	Round erythematous superficial ulcers	Increase in procalcitonin, CRP, ALT, AST, eosinophils	ABS,	Cure
					SS,	
					LZD	
5 (13)	6 years	Male	Papules, rashes, erythematous papules, pustules	–	MTX,	Cure
					SS	
6 (18)	4 years and 10 months	Female	Erythema, ulcers, swollen lymph nodes	Increase in leukocytes, lymphocytes, neutrophils	SS,	Cure
					ABS,	
					KTF,	
					Lora	
Present case	3 years and 9 months	Female	Erythematous ulcers, mucosal involvement	Elevated monocytes, lymphocytes, CRP, and serum amyloid A, Positive staphylococcus aureus subsp. aureus, Scattered CD3^+^, CD4^+^, CD8^+^, CD56^+^, S-100 and CD68, polyclonal TCR gene rearrangement	CMZ,	Cure
					MTX,	
					IVIG,	
					SS	

ACV, Acyclovir; IVIG, intravenous immunoglobulins; BAS, basiliximab; MTX, methotrexate; LZD, linezolid; SS, systemic steroids; ABS, antibiotics; LZD, linezolid; DDS, dapsone; KTF, Ketotifen; VCR, Vincristine; Lora, Loratadine; CRP, C-reactive protein.

The clinical presentation of this case aligns with other reported instances of febrile ulceronecrotic Mucha-Habermann disease (FUMHD) in preschool-aged children (3–6 years), fulfilling the diagnostic criteria proposed by Nofal et al. (4), which include: (1) consistent clinical and histopathological features; (2) variable features present in some patients. In addition to fever, acute ulceronecrotic lesions, rapid progression, and occasional mucosal involvement, flexural accentuation has been identified as another significant variable feature for diagnosing febrile ulceronecrotic Mucha-Habermann disease (19).

### 3.2 Key points for clinical and pathological differential diagnosis

FUMHD must be distinguished from other severe cutaneous and mucosal reactions, particularly Stevens-Johnson syndrome (SJS)/toxic epidermal necrolysis (TEN) and severe erythema multiforme (EM major), especially when FUMHD presents as 1–6 cm target-shaped lesions with central ulceration and peripheral vesicles. As emphasized by Shah et al. (11), the differential diagnosis depends on three key aspects: disease course (chronic vs. acute): This case presented with a 5-month relapsing-remitting course, in stark contrast to the acute onset of SJS/TEN (within 4 weeks of drug exposure) (11) and the self-limiting nature of EM major (duration of 2–4 weeks, with a mean of 15.9 days) (12). Histopathology (focal necrosis vs. full-thickness necrosis): Biopsy revealed focal necrosis with leukocytoclastic vasculitis, rather than the full-thickness epidermal necrosis and widespread keratinocyte apoptosis characteristic of SJS/TEN (11), or the vacuolar interepidermal dermatitis typical of EM major (12). Triggers and Nikolsky sign: This case lacked the drug triggers typical of SJS/TEN and did not exhibit Nikolsky sign (11). Comprehensive infection screening was negative; however, the patient's medical records did not document specific PCR test results for herpes simplex virus (HSV), varicella-zoster virus (VZV), or Mycoplasma pneumoniae. Nevertheless, the prolonged course of illness and histopathological features support the exclusion of infection-induced EM major (12). Furthermore, T-cell clonality analysis provided additional diagnostic evidence. Although clonal T-cell populations may be present in SJS/TEN, this case exhibited a specific immunophenotype (CD3+, CD8+, CD56+, TIA1−; TCR gene rearrangement was polyclonal at admission), consistent with the lymphoproliferative patterns described in the literature for FUMHD.

In this review, the cases mainly presented with generalized polymorphous skin eruptions, including erythematous patches, hemorrhagic macules, necrotic-crusted papules, as well as cutaneous ulcers and pustules. Some lesions coalesced into plaques, commonly seen on the face, trunk and extremities. The present case showed concurrent perioral ulcers and genital mucosal erosions. Wu et al. reported a case presenting with perioral ulcers, while another case exhibited both perioral ulcers and non-ulcerative white plaques on the genitalia (16, 18). Furthermore, all pediatric patients demonstrated persistent high-grade fever, with body temperatures ranging between 38 and 40 °C. Among the seven reported cases, four manifested lymphadenopathy, with one case showing additional symptoms of epileptic seizures and lymph node involvement alongside high fever (16).

The exact etiology and pathogenesis of FUMHD remain unclear. Among these 7 pediatric cases, no definitive causative pathogens were specifically identified. Although some cases reported a history of upper respiratory tract infections (14) or tested positive for Mycoplasma pneumoniae IgM antibodies (15), and the current patient had bronchopneumonia during hospitalization. However, the direct correlation between these factors and the disease has not been conclusively established.

Cutaneous and blood cultures in FUMHD patients predominantly identify Staphylococcus aureus and pseudomonas aeruginosa as the primary bacterial pathogens (16, 17). Skin secretion cultures from this current case identified Staphylococcus aureus subsp. aureus, which aligns with the findings reported by Tang et al (10). Bacterial infections may exacerbate the disease progression, elevating the risks of septicemia and mortality (4, 20). However, the precise mechanisms by which infectious triggers exacerbate FUMHD at cellular and cytokine levels remain poorly understood. This pathophysiological uncertainty underscores the critical need for rigorous infection prevention and control in pediatric FUMHD cases to mitigate disease progression.

Orenstein et al. (15) revealed that FUMHD is characterized by infiltration of mature, morphologically bland CD3^+^/CD8^+^ T cells with epidermotropism, which differs from the atypical lymphocytes, prominent epidermotropism, and polymorphous infiltrates typically seen in cutaneous involvement by T-cell lymphoma or leukemia. Consistent with this report, our case also exhibited infiltration of mature CD3^+^/CD8^+^/TdT^−^ T cells with epidermotropism, along with abnormal infiltration of CD4^+^ and CD56^+^ T-cell subsets.

Emerging evidence suggests that FUMHD represents a lymphoproliferative disorder characterized by dynamic T-cell clonality patterns (2). Cozzio et al. (21) proposed that clonal FUMHD should be classified as a distinct cutaneous T-cell lymphoma entity, with T-cell clonality potentially correlating with disease severity. Early studies detected polyclonal T-cell populations in FUMHD patients (20, 22), a finding consistent with our current case result. Notably, emerging evidence reveals dynamic changes in T-cell clonality during FUMHD progression. For instance, a reported pediatric case with pre-existing leukemia initially exhibited monoclonal T-cell expansion, which later transitioned to polyclonal patterns upon clinical improvement (15). This observation suggests that clonal evolution in FUMHD may correlate with disease activity, where polyclonality could serve as a potential indicator of favorable prognosis.

### 3.3 Therapeutic strategies for refractory FUMHD

Although the patient in this case achieved remission with conventional immunosuppressants, cases of refractory FUMHD may require targeted biological therapy. Orenstein et al. (15) reported a case of severe “Muhar-Habermann-like” ulcerative necrotizing dermatosis associated with T-cell acute lymphoblastic leukemia (T-ALL), in which was refractory to methotrexate and corticosteroids but responded to paliximab (an anti-CD25 antibody), suggesting that anti-IL-2 receptor therapy is a viable option in the context of elevated interleukin-2 (IL-2) levels. Furthermore, Kreuter et al. (23) reported a case of refractory FUMHD in an adult who achieved complete remission after two courses of infliximab (a tumor necrosis factor-α inhibitor), suggesting that tumor necrosis factor-α blockade may be effective in treatment-resistant cases (evidence level: case report). Although these biologics were not used in the present case, they represent important salvage treatment options when standard immunosuppressive therapy fails or is contraindicated, particularly for cases with underlying lymphoproliferative features.

We acknowledge that the case described by Orenstein et al. represents a “Muha-Habermann-like” rash in the context of T-ALL rather than classic FUMHD. However, we included it in our treatment considerations for the following reasons: (1) histopathological features (epidermal affinity of mature CD3+/CD8+ T cells) and clinical presentation (febrile ulcerative necrotic plaques) are indistinguishable from those of FUMHD; (2) the successful use of baliximab provides key evidence for the efficacy of anti-CD25 therapy in T-cell atypic ulcerative necrotic dermatoses, supporting the concept of FUMHD as a lymphoproliferative disorder.

The patient initially received systemic corticosteroids and antibiotics with poor response, showing no significant improvement in overall condition, while ulcerated necrotic lesions progressed with high fever and severe pain. Five days later, an intensified regimen incorporating pulse prednisolone therapy, MTX, and IVIG was initiated. Within two weeks, fever resolved, new lesions ceased to develop, and existing lesions began to heal. Although no consensus exists on first-line therapy for FUMHD, current management strategies for this rare disorder include systemic corticosteroids, MTX, cyclosporine, dapsone, IVIG, and adjunctive traditional Chinese medicine (13, 18). In this case, MTX monotherapy showed limited efficacy, but marked improvement was achieved after combining IVIG and high-dose corticosteroids, aligning with reported therapeutic responses in similar cases (16). Given the heterogeneous treatment responses and frequent use of combination therapies in FUMHD, definitive conclusions about a single treatment efficacy remain challenging.

## 4 Conclusion

FUMHD is characterized by high fever and progressive cutaneous ulceration as its hallmark manifestations, frequently accompanied by systemic involvement and infectious complications. Existing clinical evidence supports multiple therapeutic strategies for FUMHD management, including infection control, immunomodulation, and biologic agents, supplemented by supportive care. Methotrexate serves as the first-line therapy, while IVIg and basiliximab may be considered for refractory cases. Further researches are required to elucidate the pathogenesis of FUMHD, which will facilitate development of targeted therapies and improve early detection and management.

## Data Availability

The original contributions presented in the study are included in the article/Supplementary Material, further inquiries can be directed to the corresponding author.
